# Suitability of Data-Collection Methods, Tools, and Metrics for Evaluating Market Food Environments in Low- and Middle-Income Countries

**DOI:** 10.3390/foods10112728

**Published:** 2021-11-08

**Authors:** Selena Ahmed, Gina Kennedy, Jennifer Crum, Chris Vogliano, Sarah McClung, Collin Anderson

**Affiliations:** 1 Department of Health and Human Development, Montana State University, Bozeman, MT 59717, USA; 2 USAID Advancing Nutrition, Arlington, VA 22202, USA; gina_kennedy@jsi.com (G.K.); jennifer_crum@jsi.com (J.C.); chris_vogliano@jsi.com (C.V.); smcclung@advancingnutrition.org (S.M.); collin_anderson@jsi.com (C.A.)

**Keywords:** food environments, markets, assessments, methods, tools, metrics, food system, healthy diets

## Abstract

Globalization is transforming food environments in low- and middle-income countries (LMICs) with implications for diets and nutrition. However, most food-environment assessments were developed for use in high-income countries. We evaluated the suitability of 113 data-collection assessments (i.e., methods, tools, and metrics) for eight dimensions of informal and formal market food environments for diverse contexts of LMICs. We used a scoring exercise and a survey of experts (*n* = 27). According to the scoring exercise, 10 assessments (8 methods, 1 tool, and 1 metric) were suitable without modification for informal markets. Suitability for formal markets was markedly higher, with 41 assessments (21 methods, 14 tools, and 6 metrics) found suitable without modification. Experts considered availability, accessibility, price, and affordability the most important dimensions of market food environments to evaluate in LMICs. Market-basket analysis and vendor audits (which include inventories) were ranked as the most suitable methods to assess multiple dimensions of market food environments, including availability, price, affordability, vendor and product characteristics, marketing, and regulation. Gaps in relevant assessments were found for convenience and desirability. Results demonstrate the need for the development, adaptation, and validation of assessments relevant for informal markets in a diverse range of LMIC contexts to support diets, nutrition, and health globally.

## 1. Introduction

Food environments in low- and middle-income countries (LMICs) are changing, spurred by the globalization of food systems [1] and the increased diversity (healthy and unhealthy) of foods available in both formal and informal markets [2]. Foods purchased from markets make up a growing percentage of diets in urban and rural areas and across wealth brackets [3]. For example, poor rural and urban households in East and Southern Africa purchase 48% of their food from markets, whereas middle-class households purchase approximately 60–80% [3]. Shifts in food access and dietary choices in many LMICs have contributed to dietary and nutrition transitions [4,5,6,7] toward increases in, and increased risk of, diet-related noncommunicable diseases [8,9,10,11]. Health outcomes associated with the nutrition transition are recognized to affect economic and human development [12]. Given that poor diets are a leading contributor to the global burden of disease [13], understanding the factors that influence food access and food choices is critical to improving nutrition and health outcomes globally.

Within the food system, the food environment is the nexus between food supply and demand and is increasingly recognized as a primary driver of dietary patterns [9,14,15,16]. There are various types of food environments, including natural and built food environments [14]. Natural food environments include wild and cultivated food environments, and built food environments include informal market food environments (e.g., open-air markets such as farmer’s markets, wet markets, street vendors, kiosks, mobile vendors) and formal market food environments (e.g., supermarkets, hypermarkets, online vendors, restaurants) [9,14,15,16]. Informal market food environments are often not regulated through formal governance structures [17], and are supported by traditional and modern-to-traditional food supply chains [14,18]. Formal market food environments are those that are regulated through formal governance structures where sellers can publicly advertise their locations and prices, and are supported by modern and traditional-to-modern food supply chains. Some types of market food environments, such as farmer’s markets, may be classified as formal market food environments or informal markets depending on the governance structure and the nature of supply chains. In LMICs, informal market food environments are generally found in rural areas, whereas mixtures of formal and informal market food environments are more prevalent in urban areas. As more households purchase foods to meet dietary needs, market food environments have greater influence on consumers’ food choices, nutrition, and health outcomes [3,19].

Multiple researchers have proposed frameworks to characterize food environments in LMICs [9,14,15,20]. Although the frameworks have many similarities, they also place emphasis on one or more divergent areas. For example, the framework developed by Downs et al., (2020) places additional emphasis on the wild, cultivated, and market food environments and includes the dimension of sustainability. The Turner et al., (2018) framework categorizes food environments based on external and personal domains, which each include four dimensions. The external domain includes availability; price; vendor and product properties; and marketing and regulation. The personal domain includes accessibility, affordability, convenience, and desirability. Toure et al., (2021) emphasize market food environments and propose evaluating the position of food retail within a defined geographic area and characteristics of foods encountered inside markets and other retail locations. Despite the development of frameworks for characterizing food environments globally [14,16], most research has focused on assessing only one or a few dimensions of the food environment [14,21].

Despite advances in the development of data-collection assessments (including methods, tools, and metrics) to evaluate food environments over the past 20 years [15], most assessments have been developed, conducted, and validated in high-income country contexts, where food environments vary dramatically from the diverse range of those found in LMICs [14,21]. For example, consumers in high-income countries primarily access formal market food environments, including supermarkets, restaurants, and fast-food chains, many of which are open 18–24 h per day and are accessed using vehicles and paved roads. In contrast, rural households in LMICs more commonly procure foods from natural food environments and informal market food environments, such as open-air markets, that have limited schedules and highly seasonal food offerings [14,22]. Poor road conditions, especially during rainy seasons, and limited use of motorized vehicles further affect market accessibility in rural areas. These differences underscore the importance of understanding the suitability of existing food-environment methods, tools, and metrics for informal and formal markets outside high-income settings. The lack of appropriate assessments for the diverse contexts of LMICs limits monitoring and evaluation of factors that influence food access at point of procurement.

Although recent frameworks [14,16,21] demonstrate advances in assessment considerations for evaluating food environments in LMICs, there remains a knowledge gap regarding the suitability of different assessments for use in diverse contexts. The purpose of this study was to evaluate the suitability of data-collection assessments (including methods, tools, and metrics) for multiple dimensions of the external and personal domains of market food environments in informal and formal markets in LMICs. We recognize that tremendous variation exists across LMICs based on infrastructure, socio-cultural factors, economic development, rurality, politics, topography, agro-climatic zones, and other factors that impact food environments and food access. Our study was intended to evaluate assessments that are suitable across the diverse range of contexts of LMICs, including those where informal markets are prevalent. Our study objective was addressed using a scoring exercise to evaluate the suitability of food-environment assessments and a structured survey of global experts to elicit input on experiences and perspectives regarding food-environment data collection in LMICs. It is expected that study findings will inform the monitoring and evaluation of market food environments in LMICs through identification of suitable data-collection assessments and inform the development and validation of new assessments where gaps currently exist.

## 2. Materials and Methods

The widely accepted Turner et al., (2018) framework [16] was chosen as the organizational structure for our research because it builds on previous food-environment frameworks (HLPE 2017) and has been included in landmark reports such as the 2019 State of Food Insecurity in the World [23]. The Turner et al., (2018) framework categorizes external and personal food environments based on the external dimensions of availability, price, vendor and product properties, and marketing and regulation, along with personal dimensions of accessibility, affordability, convenience, and desirability.

### 2.1. Literature Review

The research team conducted a literature search using the key term “food environment” to compile a list of existing data-collection assessments defined as either a method, tool, or metric for evaluating the food environment. The PubMed, Agricola, USAID Development Experience Clearinghouse (for papers on market information systems [MISs]), Google Scholar, and Web of Science databases were searched. The inclusion criteria were: (1) a focus on market food environments; (2) available in English; and (3) published after 1998. The team then used a snowballing approach of referencing additional articles from citations in the identified articles, including recently published reviews on food-environment assessments [14,15,16,24].

### 2.2. Assessment Categorization

**Figure 1:**
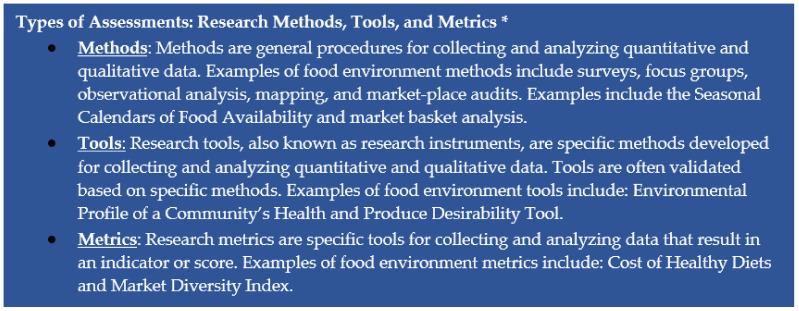
Definitions of assessment types: Methods, tools, and metrics. * This classification system is not always distinct with specific measures falling neatly between tools and metrics.

The assessments were categorized as either a method, tool, or metric (See Figure 1 for definitions and examples) and assigned to one or more of the eight dimensions of the external and personal domains of the Turner et al., (2018) food-environment framework. For this study, methods were defined as general procedures for collecting and analyzing quantitative and qualitative data such as surveys, focus groups, observational analysis, mapping, and audits. Tools were defined as research instruments specifically developed to collect and analyze quantitative and qualitative data, often based on a specific method. Metrics were defined as parameters, indexes, and indicators used to measure, compare, or track performance or outcomes.

Categorizing the data-collection assessments by the external and personal domains of the Turner et al., (2018) food-environment framework resulted in a list of 74 assessments to evaluate the external domain and 39 assessments to evaluate the personal domain, with several assessments assigned to more than one food-environment dimension (Supplementary Table S1. Of the eight food-environment dimensions, availability had the largest number of methods (*n* = 11) followed by vendor and product characteristics (*n* = 9) and convenience (*n* = 7). For tools, availability had the most (*n* = 15) followed by vendor and product characteristics (*n* = 8) and accessibility (*n* = 7). For metrics, price had the largest number (*n* = 7) followed by affordability (*n* = 6).

### 2.3. Suitability Scoring Exercise

Each of the four research team members scored the categorized list of data-collection assessments and accompanying descriptions for suitability in LMICs in: (1) informal market settings, (2) formal market settings, and (3) both informal and formal market settings. Scores of 0 (indicating not appropriate or would require major modification), 1 (could be appropriate with minor modification), or 2 (appropriate as is) were assigned to each assessment. For example, food-environment assessments developed in a high-income country and focused on attributes of supermarkets such as shelf placement were scored as 0 for use in an informal market setting, 2 for use in a formal market setting, and 0 for both informal and formal settings. Given that food vendors may be mobile and/or not listed in directories, assessments that examined density of food vendors were assigned a score of 1 for use in an informal market setting, 2 for use in a formal market setting, and 1 for both informal and formal settings. Assessments developed, piloted, and validated in rural and urban LMIC contexts were scored as 2.

The scores from each research team participant were compiled and averaged to develop Suitability Scores for informal market settings, formal market settings, and combined informal and formal market settings. The standard deviation of participant scores was calculated to evaluate variation in scoring as a measure of inter-rater reliability; discrepancies greater than 0.5 points were discussed as a team. All assessments that received an average Suitability Score of 1.5 or greater for combined informal and formal markets were determined to be suitable for use in LMIC contexts, either in the current form or with minor modification.

### 2.4. Semi-Structured Survey with Global Experts

Assessments that received Suitability Scores of 1.5 or greater were included in a survey instrument sent to global experts with experience working in LMICs. The survey consisted of 20 questions grouped in four parts (Supplementary Table S2). The survey was approved for exempt status concerning participation of human subjects by the Institutional Review Board (IRB) of John Snow, Inc. (IRB no. #20-25E). Fifty-one experts in food environments, food security, and nutrition were identified based on their published work and presentations during international conferences and meetings. Some of the experts included known contacts working on food-environment research or projects. The group of identified experts received an online version of the survey; of these, 27 (>50%) completed the survey. The experts identified themselves as researchers (48%), professors (33%), other (11%), and field practitioners (8%).

The survey consisted of four parts: (1) informed consent; (2) background information on expert’s area of work, food-environment dimensions generally measured, and food-environment dimensions perceived as most important; (3) practices and perceptions concerning food-environment methods, including characteristics for deciding if a specific food-environment assessment is suitable in LMICs; and (4) practices and perceptions regarding food-environment tools and metrics. Experts were requested to rate the suitability of methods for evaluating market food environments in rural areas (where informal market food environments are often prevalent) in LMICs as “not suitable,” “somewhat suitable,” and “very suitable” (survey part 3, questions 8–13; Supplementary Table S2). In part 4, the tools and metrics identified to evaluate the eight food-environment dimensions were rated on a five-point Likert scale (survey part 4, questions 15–18; Supplementary Table S2). Each tool or metric was evaluated for the following attributes: (1) suitability (a score of 5 was “most suitable”); (2) level of training (a score of 5 indicated relatively high level of training); (3) resource intensity; and (4) “translatable/operationalizable.” Experts were then asked to provide examples of perceived gaps in food-environment assessments applicable in LMIC settings.

Experts often are more familiar with one or two domains of the food environment; therefore, we developed three versions of part 4 of the survey to score tools and metrics for (1) availability and accessibility; (2) marketing, vendor and product characteristics, convenience, and desirability; or (3) prices and/or affordability.

Survey data were analyzed for frequency of responses. Importance Scores were calculated by averaging the weighted rankings of the importance of each of the eight dimensions of the food-environment framework for understanding rural contexts in LMICs for influencing diets and nutrition. The responses were equally weighted based on the ranking order given by the experts. Two sets of Importance Scores were calculated; the first set was based on the experts’ ranking of the food-environment dimensions in their own practice (survey part 2, question 5; Supplementary Table S2), and the second set was based on responses when taking the perspective of a program officer of an agency such as USAID to inform evidence-based approaches to improving food security (survey part 2, question 6; Supplementary Table S2). Results from the five-point Likert-scale questions rating tools and metrics (survey part 4, questions 15–18; Supplementary Table S2) were tabulated into a weighted Feasibility Score ranging from 0 to 100 that comprised the following: (1) average ratings of suitability (weight, 42.85%); (2) inverse of the average ratings of level of training (weight, 14.29%); (3) inverse of the average ratings of resource intensity (weight, 14.29%); and (4) average ratings of translatable/operationalizable (weight, 28.57%). Totals were proportionately multiplied to a 100-point scale. Feasibility Scores from 0 to 33% were considered low, from 34 to 67% were considered moderate, and from 68 to 100% were considered high.

## 3. Results

### 3.1. Suitability Scores

**Figure 2:**
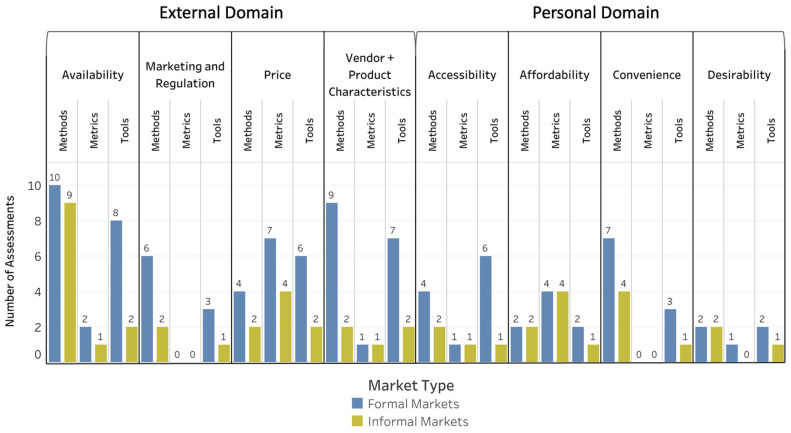
Market Food-Environment Assessments Suitable for LMICs with Minor Modification. Number of methods, tools, and metrics found suitable based on the scoring exercise with minor modification in LMICs.

**Figure 3:**
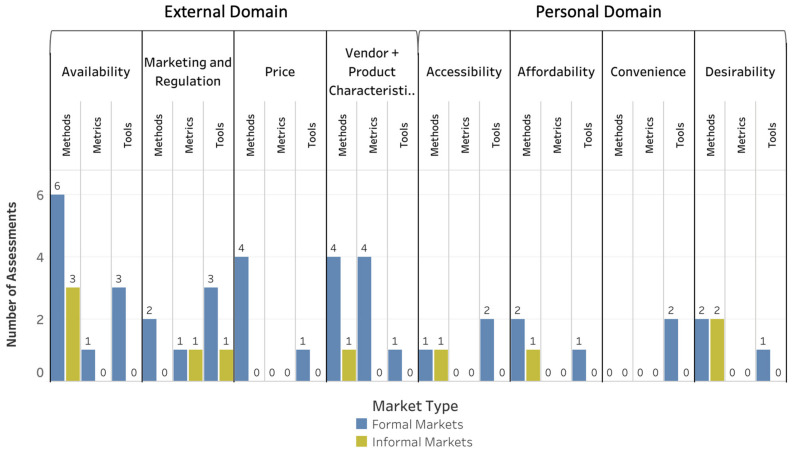
Market Food-Environment Assessments Suitable for LMICs without Modification. Number of methods, tools, and metrics found suitable based on the scoring exercise without modification in LMICs.

According to results of the suitability scoring exercise, 47 (25 methods, 11 tools, and 11 metrics) of the 113 assessments were determined to be suitable with minor or no modification (Suitability Score, ≥1.5) for informal market food environments as well as collectively for informal and formal market food environments in LMICs (Figure 2; Supplementary Table S1). Notably more assessments (*n* = 97; 44 methods, 37 tools, and 16 metrics; Figure 2) were suitable with minor or no modification for formal markets than for informal markets. Ten assessments (8 methods, 1 tool, and 1 metric) were identified as suitable without modification (Suitability Score, 2.0) for informal markets (Figure 3), and 41 assessments for formal markets were suitable in their current form (21 methods, 14 tools, and 6 metrics; Figure 3). Assessments for use in formal markets had higher Suitability Scores for both the external and personal domains (Suitability Score, 1.60 and 1.59, respectively) compared with informal markets (Suitability Scores, 0.86 and 1.15 for the external and personal domains, respectively) as well as collectively for formal and informal markets (Suitability Score, 0.92 and 1.17, respectively).

**Figure 4:**
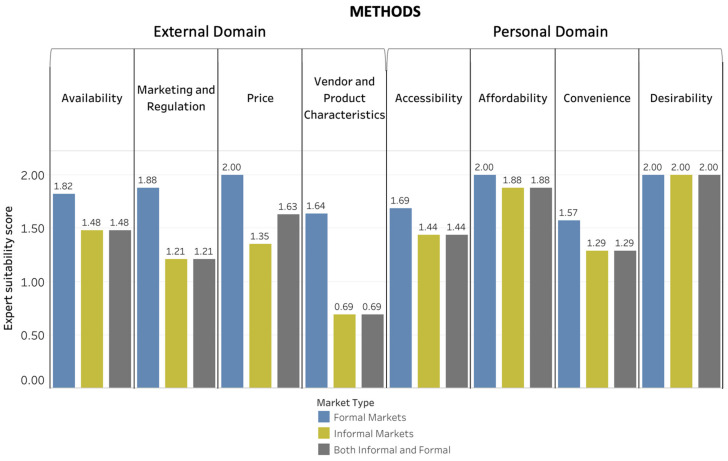
Average Suitability Scores for Food-Environment Methods. Average Suitability Scores for methods for evaluating food-environment dimensions based on the scoring exercise on a scale of 0 to 2 (0: not appropriate and/or would require major modification; 1: could be appropriate with minor to moderate modification; 2: appropriate as is).

**Figure 5:**
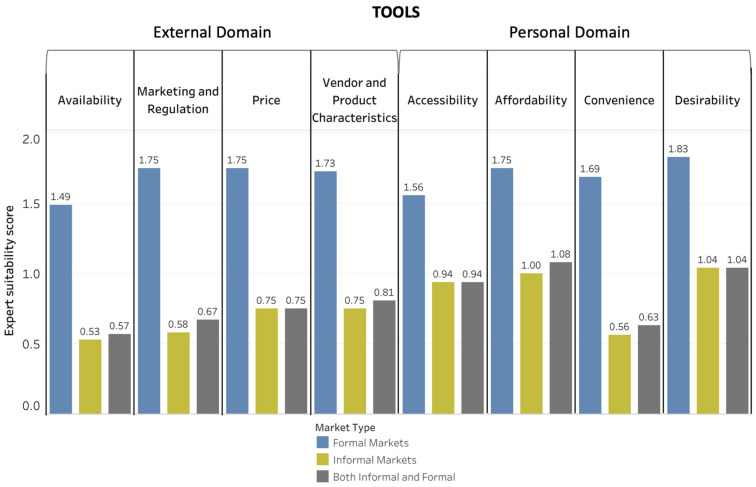
Average Suitability Scores for Food-Environment Tools. Average Suitability Scores for tools for evaluating food-environment dimensions based on the scoring exercise on a scale of 0 to 2 (0: not appropriate and/or would require major modification; 1: could be appropriate with minor to moderate modification; 2: appropriate as is).

**Figure 6:**
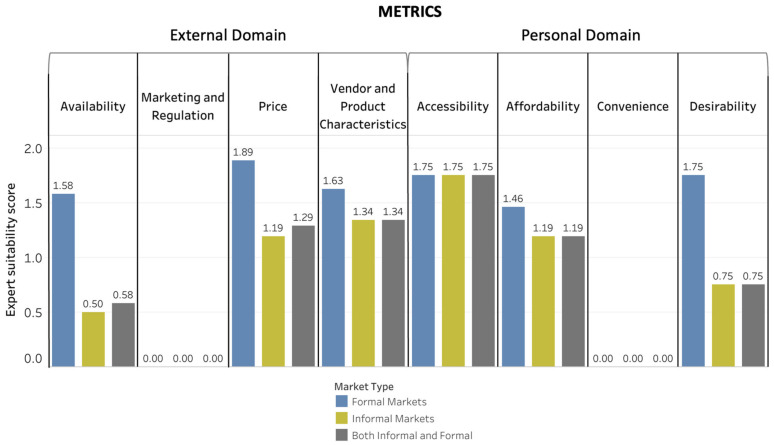
Average Suitability Scores for Food-Environment Metrics. Average Suitability Scores for metrics for evaluating food-environment dimensions based on the scoring exercise on a scale of 0 to 2 (0: not appropriate and/or would require major modification; 1: could be appropriate with minor to moderate modification; 2: appropriate as is).

Figure 4, Figure 5 and Figure 6 depict the Suitability Scores for each dimension of the food environment for use in informal markets, formal markets, and collectively for both types of markets for methods (Figure 4), tools (Figure 5), and metrics (Figure 6). Supplementary Table S1 provides a brief description and reference(s) for each of the data-collection methods, tools, and metrics identified as suitable for use in LMICs either with no or minor modification needed.

For methods, price was the food-environment dimension with the highest Suitability Score (1.63) for both informal and formal markets, whereas desirability was the dimension in the personal domain with the highest Suitability Score (2.0). Vendor and product characteristics had the highest Suitability Score for tools (0.81) and metrics (1.32) in the external domain of the food environment. Affordability was the food-environment dimension in the personal domain with the highest Suitability Score for tools (1.04), and accessibility had the highest Suitability Score for metrics (1.75).

The food-environment dimensions in the external domain with the most assessments rated as suitable without modification for use in both informal and formal markets were availability for methods (three methods) and vendor and product characteristics for both tools and metrics (one tool and one metric). The food-environment dimensions with the most personal domain assessments rated as suitable without modification for use in both informal and formal markets were desirability for methods (two methods) with no suitable tools or metrics.

### 3.2. Semi-Structured Survey with Global Experts

When asked the type of food environment they most commonly assess, experts responded informal market (78%), formal market (63%), cultivated (56%), and wild (33%) food environment. When asked about the most commonly measured dimensions, responses were availability (89%) price (74%), desirability (74%), vendor and product characteristics (67%), accessibility (67%), convenience (63%), and sustainability (52%).

**Figure 7:**
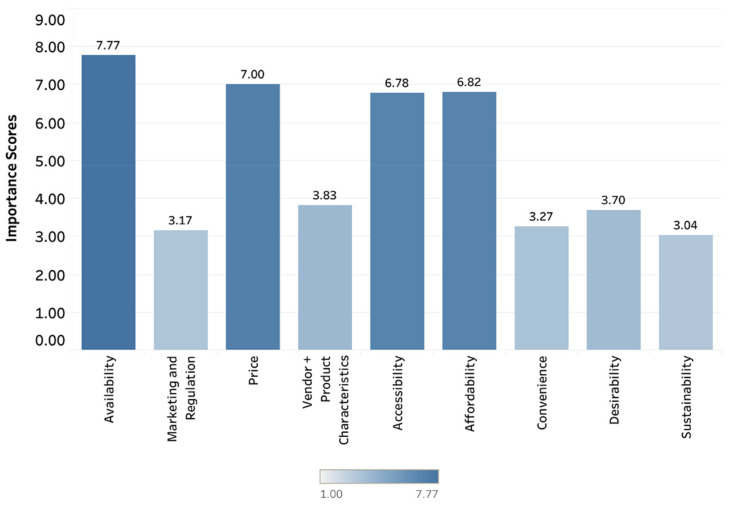
Importance Scores for Food-Environment Dimensions. Importance Scores based on the surveyed experts’ weighted rankings of the dimensions of the food-environment framework that they perceive as the most important to measure for understanding rural contexts in LMICs for influencing diets and nutrition from the perspective of programming.

Experts perceived availability (Importance Score, 7.71), accessibility (Importance Score, 7.04), affordability (Importance Score, 6.92), and price (Importance Score, 6.42) as the dimensions most important for understanding rural contexts in LMICs for influencing diets and nutrition. When considering a program perspective, the Importance Score order (from highest to lowest) differed from that of the experts’ personal perspective as follows: availability (7.77), price (7.0), affordability (6.82), and accessibility (6.78) (Figure 7).

The surveyed experts identified a range of characteristics they value when identifying suitable food-environment assessments for LMICs. The characteristics reported by 50% or more of the experts include (in order of prevalence): (1) the assessment captures multiple dimensions of the food environment (95%); (2) culturally relevant (91%); (3) context specific (77%); (4) appropriate for diverse types of food environments (68%); (5) easy to implement (68%); (6) results in findings that are translational (64%); (7) cost-effective (64%); (8) validated (59%); and (9) rapid (50%). The following characteristics were reported by less than half of the surveyed experts for identifying suitable food-environment assessments for LMICs: (1) the assessment is objective (45%) and (2) scalable (23%).

Table 1 lists the methods for each food-environment dimension experts rated as very suitable, somewhat suitable, or not suitable for a rural context in LMICs, where informal market food environments are often prevalent. One or more methods were categorized as very suitable for each dimension of the food environment, including five methods for availability, two for price, one for vendor and product properties, one for marketing and regulation, two for accessibility, one for affordability, two for convenience, and one for desirability. The method of vendor audit [25] was found very suitable for evaluating multiple food-environment dimensions, including availability, price, vendor and product properties, and marketing and regulation. Market-basket analysis [26] was either very suitable or somewhat suitable for evaluating multiple food-environment dimensions, including availability, price, vendor and product properties, marketing and regulation, accessibility, convenience, and affordability. Consumer surveys [25,27] were either very suitable or somewhat suitable for evaluating every food-environment dimension.

Table 2 lists the Feasibility Scores for each of the tools and metrics for evaluating market food environments in LMICs in order of highest to lowest score for each dimension. In addition, Table 2 lists the ratings of the four components composing Feasibility Scores, including suitability, level of training, resource requirements, and translational/easily operationalized. Although market information systems received the highest Feasibility Score for availability and accessibility, it was rated by the experts as requiring relatively high levels of training and resources. Despite the dimensions of price and affordability having the most assessments, the Feasibility Scores for each assessment were less than 61%. For vendor and product characteristics and marketing and regulation, Environmental Profile of a Community’s Health (EPOCH) received the highest Feasibility Score; this assessment was rated to require a moderate level of training and resource requirements. American Time Use Survey for assessing convenience received the lowest Feasibility Scores of all the assessments for all food-environment dimensions. The Produce Desirability (ProDes) Tool was the only tool/metric evaluated for desirability; it received a moderate Feasibility Score.

Several of the surveyed experts reported tools and metrics for evaluating food environments that were not in the survey; the most prevalent of these was the Market Diversity Index. This metric was not included in the survey because it was under review for peer-reviewed publications when we conducted our search for assessments to include in the scoring exercise and survey. From experts’ responses, key themes of what is needed to enhance food-environment assessments in LMICs emerged. These included ensuring food-environment assessments suitable for LMICs are available and developing guidance protocols, best practices, and training on suitable assessments.

## 4. Discussion

Findings highlight the need for modification of existing assessments and the development of new assessments for evaluating dimensions of the informal market food environment in LMICs. The suitability scoring found notably more assessments as suitable without modification for formal market food environments than for informal market food environments. Availability, accessibility, price, and affordability were considered by the global experts surveyed as the most important dimensions of market food environments to evaluate in LMICs. Market-basket analysis and vendor audits (including inventories) were the most suitable methods to assess the food-environment dimensions of availability, price, vendor and product characteristics, marketing and regulation, and affordability. Consumer surveys [32] were ranked as “very” or “somewhat” suitable for all eight food-environment dimensions. Feasibility Scores were low to moderate for most tools and metrics included in this review, except for MISs and EPOCH [33]. Table 3 lists assessments recommended by our research team for evaluating food environments in LMICs along with considerations for adapting various assessments.

The assessments identified as suitable for evaluating each food-environment dimension have varying levels of data, resource, and skill requirements. A method may be deemed suitable while needing to be developed into a data-collection tool or metric specific for informal and formal markets in LMICs. Many methods lack a corresponding tool including consumer surveys/interviews, vendor audits, mapping, market-basket analysis, and photo-elicitation. The development of relevant tools and metrics from available methods is one of the main assessment needs we identified.

The scoring exercise found low suitability of assessments for use in informal market food environments in LMICs, especially for convenience and desirability, and points to a need for adaptation of existing assessments or development of new ones. For example, the Nutrition Environment Measurement for Stores was developed for a USA context with food items benchmarked based on American diets [34]. Other assessments had low suitability in informal markets because of resource requirements including technology and access to secondary data, such as national data and directories of food vendors. For example, the Consumer Price Index for Food was scored as requiring major modification for use in informal markets, due to limitations in accessing price data. Many of the assessments that were found as suitable or feasible, from the expert survey, would need to be modified to be relevant for LMIC contexts.

Survey findings that availability, accessibility, price, and affordability were the most important dimensions of market food environments to evaluate in LMICs correspond with the recent literature. Specifically, the food-environment frameworks of Herforth and Ahmed (2015), HLPE (2017), Turner et al., (2018), and Downs et al., (2020) all include the dimensions of availability and/or accessibility as well as price and/or affordability. However, the Herforth and Ahmed (2015) and Downs et al., (2020) frameworks only include the external domain of the food environment; they do not view the personal domain as part of the food environment but rather the way individuals interact with the food environment. The finding that availability is the most important dimension of market food environments to evaluate in LMICs is further aligned to the finding that multiple methods were found suitable for this dimension.

Market-basket analysis and vendor audits, the two methods found most suitable to assess multiple food-environment dimensions in LMICs, are suitable because they can be modified for observing different types of market food environments and a range of food groups appropriate for LMICs. Research and development are needed to modify existing audits to include culturally relevant food groups (e.g., those based on national FBDGs) as well as other characteristics, such as vendor types. Other suitable food-environment methods for LMICs include market mapping [35], Seasonal Calendars of Food Availability [36], and photo-elicitation [37]. Research is also called for to develop a classification system of types of informal market food environments for the market mapping assessment to be more relevant for diverse LMIC contexts.

Despite MISs receiving the highest Feasibility Score for evaluating availability, shortcomings of this assessment for use in informal market settings were noted. These included access to technology and secondary data, such as national price data on various foods, as well as questionable relevance of national price data for diverse contexts within an LMIC. In addition, an MIS may be characterized as a method with metrics, such as Cost of Healthy Diets, being a specific type of MIS.

MFDI was not included in the survey, because the peer-reviewed paper about this metric was not published at the start of this study. However, some experts surveyed noted it as a suitable metric, and it is increasingly being implemented in LMICs. Thus, MFDI is included by our research team as a recommended metric to evaluate market food environments in LMICs (see Table 3). MFDI has the potential to be suitable in diverse contexts because it can be benchmarked to multiple food group classification systems to calculate dietary diversity scores such as the Food and Agriculture Organization of the United Nations Guidelines for Measuring Dietary Diversity Scores (16 food groups) and the Minimum Dietary Diversity for Women (MDD-W; 10 food groups), both of which are extensively used in LMICs for evaluating diets.

An adapted version of the HEI of Food Supply is included by our research team as a recommended metric to evaluate the availability of foods that are aligned to quantitative FBDGs in market food environments in LMICs, based on the Feasibility Score from the survey responses (see Table 3). The Healthy Eating Index (HEI) of Food Supply, which is benchmarked to national food-based dietary guidelines (FBDGs) in the United States can be modified to be benchmarked to quantitative FBDGs that are culturally relevant. In addition, because the HEI of Food Supply requires quantitative inventories of food weights that may be difficult to procure in an informal market setting, modification is required to collect these data via vendor interviews as published lists may not be available.

Vendor audit and market-basket analysis, the methods rated as very suitable for measuring food price and affordability, are the basis of several metrics including Cost of a Healthy Diet (CoHD; formerly called Cost of Recommended Diet) [38] that received the highest Feasibility Score for the dimensions of price and affordability. CoHD is benchmarked to quantitative FBDGs, and thus includes all food groups recommended as part of selected quantitative FBDGs rather than only staple foods. However, CoHD faces the same challenge as the HEI of Food Supply of the lack of quantitative national FBDGs in many LMICs. In addition, because CoHD was developed for the national level with national food price data, adaptation is needed for food environments. This adaptation requires collecting food prices at the market level through vendor interviews prices of food items based on food groups aligned to the selected quantitative FBDGs. This adapted approach using interviews has financial and labor-resource requirements and requires evaluate of its’ feasibility and validity in LMICs. Future collection of food price data in market food environments may rely on emerging technology such as mobile applications for crowd sourcing food price data or directly sourcing it from market vendors.

Vendor audits, the method rated as very suitable for measuring vendor and product properties and marketing and regulation, is the basis of the EPOCH tool, which had the highest Feasibility Scores for these food-environment dimensions. The EPOCH instrument was designed to be suitable in diverse cultural, socioeconomic, and regional (urban and rural) settings across different communities, regions, and countries. The inter-rater reliability of EPOCH was validated in 93 rural and urban communities in five countries (Canada, Colombia, Brazil, China, and India) and demonstrated excellent reliability [33]. The EPOCH tool has two parts: an objective environmental audit tool to record physical aspects of the environment and an interviewer-administered questionnaire to capture community perceptions from people living in that community [33]. Thus, the EPOCH tool assesses the external and personal dimensions of the food environment. The EPOCH tool should be adapted to include a diverse range of types of market food environments and vendor types specific to LMIC contexts.

Gaps to be addressed by additional development of tools and metrics were found for the personal food-environment dimensions of convenience, accessibility, and desirability. Previous studies [39] found a lack of standardized indicators for access, with indicators of accessibility ranging from measuring distance to a market, travel time required to reach a market, or simple presence or absence of a market. In addition to measuring the same construct of market access in different ways, attention to terrain (i.e., hilly or flat), seasonal deterioration of roads, and availability of public transportation was often a limiting factor in indicator interpretation.

Although most experts evaluated consumer surveys as suitable for measuring the personal domain, the limited tools and metrics that exist need adaptation. For example, the ProDes tool was developed and validated for rural and urban contexts in the United States based on a market basket of fruit and vegetables listed in the Nutrition Environment Measurement Survey for Stores [28]. For adaptation of the ProDes to a LMIC context, research is needed to develop a market basket of produce that is culturally relevant in each context through community engagement using interviews and free listing tasks. As ProDes only evaluates fruit and vegetables, research and development are called for to evaluate desirability of food from multiple food groups aligned to FBDG.

Findings of the lack of tools and metrics for several dimensions of the personal domain of the food environment are aligned with the discrepancy in food-environment frameworks regarding whether the personal domain is part of the food environment or rather comprises the ways individuals interact with the food environment. Regardless of the place of the personal domain, it is critical to understand the dimensions of the personal domain because these notably influence healthy diets, nutrition, and health. The overall gaps identified in available data-collection tools and metrics for the personal domain of food environments are supported by previous research with similar findings. For example, the recent review by Toure et al., (2021) highlighted that gaps remain in the completeness of available assessments for dimensions of the personal domain of the food environment, including desirability and convenience.

Given the low to moderate Suitability Scores from the scoring exercise and Feasibility Scores from the survey of experts, adaptation of existing assessments and development of new assessments should consider multiple attributes valued highly by the surveyed experts including that an assessment: (1) captures multiple dimensions of the food environment; (2) is context specific; (3) is appropriate for diverse types of food environments; (4) is easy to implement; yields findings that are translational; (5) is cost-effective; (6) is validated; and (7) is rapid. Tool and metric adaptations and developments should be pilot tested in diverse LMIC contexts to ensure suitability before wider deployment. Given the need for culturally relevant assessments for informal markets and limitations of rural settings in LMICs, future work can draw from fields that have a history of rural field research (e.g., anthropology, economics, ethnobiology) and can focus on assessments that are practical and feasible for the context. To enable and enhance the routine monitoring and evaluation of multiple food-environment dimensions, such assessments should be accompanied by guidance protocols, best practices, training materials, and data analysis templates for implementing that are free, user friendly, and centrally available with clear guidance on how to interpret and synthesize findings.

For the food-environment dimensions for which a lack of suitable tools and metrics was found, the methods identified as suitable should be used as the basis to develop tools and metrics for relevance in informal markets. Importantly, assessments scored as not suitable without major modification for informal markets have potential for use in LMICs if major modifications are conducted. For example, the Retail Food-Environment Index (RFEI) [40], which compares the density of fast-food restaurants and convenience stores with that of grocery stores and produce vendors in a locality, received a low Suitability Score. However, the concept of this tool to assess the prevalence of food vendors that sell “unhealthy food” relative to food vendors that sell “healthy food” can be adapted for informal markets. The inclusion of a modified RFEI could be especially beneficial if included in mapping activities, because it results in a quantitative output that is missing from a general mapping method. An adaptation of RFEI would require a refined typology of market food environments in LMICs as well as classification of these, because food vendors sell so-called unhealthy food and healthy food.

Although this study focused on market food environments, we recognize that wild and cultivated food environments are also important for supporting food security and nutrition in LMICs [14,41] and in Indigenous and rural communities more broadly [20] and thus should be evaluated. Notable efforts over the past decade have emphasized the importance of cultivated food environments for supporting nutrition, such as the emergence of nutrition-sensitive agriculture. Previous research has further highlighted the important role of wild food environments to support food security during food shortages, economic distress, and shocks to agrifood value chains, including the COVID-19 pandemic [20,42,43]. With approximately half of the surveyed experts evaluating cultivated food environments and a third evaluating wild food environments, research and development are required to understand the suitability of data-collection methods, tools, and metrics for wild and cultivated food environments to comprehensively understand food access and the socioecological determinants of healthy diets. Assessments for evaluating multiple types of food environments would enable characterization of the food-environment transition in LMICs that is associated with the dietary transition of foods that are ultra-processed and high in saturated fat and sugar.

In addition, while sustainability received a relatively low valuation by the experts for measurement in LMICs compared with all the other dimensions of food environments, sustainability is a critical attribute of food systems to support long-term food security [44,45,46] A lack of food-environment assessments to measure sustainability is due to food-environment frameworks other than that of Downs et al., (2020) not explicitly integrating sustainability as a dimension; rather, it is included as a vendor or product attribute. Measuring sustainability in food-environment assessments is also limited due to the complexity of working across disciplines to incorporate this dimension of food system measurement. Food production has a greater impact on biodiversity, ecosystems, and greenhouse gases than any other human activity [47] while being vulnerable to environmental resource limitations and shifts in the global climate [48]. Consumers globally are increasingly motivated to make dietary choices that are more sustainable based on environmental, socio-cultural, economic, and nutrition and health attributes of foods given the critical need to transform food systems [49]; therefore, it is critical to examine if they can make such choices in their local food environments to enhance sustainability.

The development and implementation of data-collection tools and metrics that assess the sustainability of foods in the food environment, such as the Sustainability Properties of Food in the Food-Environment Rating tool [14], can support efforts to monitor and evaluate food environments toward improving human and planetary health. This tool can complement the assessments presented in Table 3 by providing much needed data regarding sustainability attributes, including seven attributes for the ecological dimension, six attributes for the economic dimension, two attributes for the human healthy dimension, and three attributes for the socio-cultural and political dimension of sustainability [14]. However, the sustainability attributes of foods lack transparency, which makes it difficult to collect these data and signals the need for greater transparency of the sustainability of foods within the food system.

Building healthy food environments and ensuring consumers have access to healthy foods are essential steps towards realizing multiple targets outlined in the United Nations Sustainable Development Goals (SDGs) [50]. Advancing healthy food systems, food environments, and diets are critically important to multiple SDGs including SDG 1, 2, 3, 5, 10, 12, 13, 14, and 15 [51]. Evaluating food sustainability in the food environment is important for understanding how food environments contribute to multiple SDGs. In LMICs, where there are recognized gaps in official data, the inclusion of data generated through meaningful citizen science (CS) and other approaches can contribute to a more comprehensive understanding of the SDGs [52,53], including targets related to food environments.

## 5. Conclusions

As primary drivers of diets, food environments are critical to understand for improving diet, nutrition, and health outcomes globally. Monitoring and evaluating market food environments in LMICs is especially critical given the increase in foods purchased from markets, diets higher in ultra-processed foods and added sugar and salt, and the associated upsurge of comorbidities that threaten human and economic development [12]. However, monitoring and evaluating food environments in LMICs are restricted by the limited suitability of data-collection methods, tools, and metrics for informal markets. The study presented here emphasize the need for refining existing data-collection methods and associated tools and metrics and developing new assessments for monitoring and evaluating multiple dimensions of informal markets. Table 3 provides a list of recommended assessments for evaluating informal market food environments in LMICs along with considerations for adaptations. These efforts should use pilot studies in diverse market food environments to generate a set of standardized assessments with accompanying guidance protocols, training materials, best practices, and data analysis templates that are free, user friendly, and centrally available. Such materials should include clear guidance on how to interpret findings from food-environment assessments for data-driven decision-making to modify food environments. Ultimately, a set of suitable and feasible food-environment assessments would enable and enhance the routine monitoring and evaluation of the multiple dimensions of food environments by governments, civil society, and program leaders to support healthy and sustainable diets and food systems that can address the global burden of disease.

## Figures and Tables

**Table 1:** Suitability of Methods Based on Expert Ratings ^a^.

Food-Environment Dimension	Methods Rated as Very Suitable	Methods Rated as Somewhat Suitable	Methods Rated as Not Suitable
Availability	- Vendor audit (including inventories) (86%) - Participatory mapping (68%) - Seasonal calendars of food availability (64%) - Photo-elicitation (59%) - Market-basket analysis (59%)	- Consumer surveys/interviews (59%) - Free listing (50%) - Shelf-space measurements (41%)	- Directory analysis (59%)
Price	- Vendor audit (64%) - Market-basket analysis (55%)	- Consumer surveys/interviews (55%)	
Vendor and Product Properties	- Vendor audit (68%)	- Market-basket analysis (55%) - Consumer surveys/interviews (50%)	
Marketing and Regulation	- Vendor audit (68%)	- Market-basket analysis (55%) - Consumer surveys/interviews (50%)	
Accessibility	- Consumer surveys/interviews (77%) - Photo-elicitation (59%)	- Market-basket analysis (64%)	
Affordability	- Consumer surveys/interviews (59%)	- Market-basket analysis (55%)	
Convenience	- Consumer surveys (77%) - Photo-elicitation (59%)	- Market-basket analysis (64%)	
Desirability	- Consumer surveys/interviews (91%) - Sensory surveys (50%) [28]	- Consumer surveys/interviews (91%) - Sensory surveys (50%)	

^a^ Suitability of the methods rated by the experts as very suitable, somewhat suitable, and not suitable for a rural context/informal markets in LMICs for each food-environment dimension. The percentage next to each assessment indicates the percentage of experts who gave the rating of very suitable, somewhat suitable, and not suitable; methods were placed in a suitability grouping based on the most prevalent response.

**Table 2:** Tools and Metrics with Feasibility Scores for Evaluating Informal and Formal Market Food Environments in LMICs ^a^.

Food-Environment Dimension	Data-Collection Tools and Metrics	Feasibility Scores, % ^b^	Suitability Rating ^c^	Level of Training ^c^	Resource Requirement ^c^	Translational/Operationalizable ^c^
Availability and Accessibility						
	MISs	78.14	4.25	4	4	4.8
	HEI ^d^	58.54	3.5	3.67	4	3.83
	ProColor Diversity Tool	40.82	3.5	4.67	3.33	2.5
Price and Affordability						
	CoHD ^d^	60.51	3.38	4.43	3.71	3.13
	EPOCH ^d^	58.49	3.8	3.83	3.5	3.2
	Cost of Dietary Diversity [29]	56.14	3.17	4	3.2	3.67
	CoNA ^d^ [29]	53.51	3.33	4.6	4	3.67
	CotD ^d^ [30]	52.17	3.38	4.43	3.71	3.13
	OptiFood	45.8	2.89	4.57	4.29	3.11
Vendor and Product Characteristics; Marketing and Regulation						
	EPOCH	68.57	4	3	3	4
	Sustainable Dimensions Food-Environment Rating Framework	60.00	3.5	2.5	2	2.5
Convenience						
	Remoteness of population [31]	61.86	3.33	1.5	1.5	2.33
	American Time Use Survey	31.43	3	5	5	1
Desirability						
	ProDes ^d^	63.34	3.75	2.75	2.33	3

^a^ Feasibility Scores and ratings of the four components composing Feasibility Scores are presented for the various dimensions of food environments. ^b^ Out of 100%. ^c^ Score range 1–5, with 5 being the highest suitability, training, resources, or easiest to operationalize, depending on component. ^d^ HEI: Healthy Eating Index of Food Supply; CoHD: Cost of a Healthy Diet; EPOCH: Environmental Profile of a Community’s Health; CoNA: Cost of Nutrient Adequacy; CotD: Cost of Diet; ProDes: Produce Desirability Tool.

**Table 3:** Recommended Assessments and Considerations to Adapt Data-Collection Methods, Tools, and Metrics for Informal Market Food Environments in LMICs.

Food-Environment Dimension	Recommended Data-Collection Methods, Tools, and Metrics	Suitability and Considerations for Adaptation for LMICs
Availability	Methods	- Existing assessments can be modified for observing different types of market food environments and range of food items specific for LMICs. - Research in diverse rural areas in LMICs is needed to identify and develop a classification of types of informal market food environments and vendor types. - Research is needed to modify existing audits and inventories to include culturally relevant food items by food groups for frequently used lists such as the 10 food groups used for MDD-W. The mapping methodology can be complemented by the inclusion of an adaptation of Retail Food-Environment Index to have quantitative comparative data output.
	- Vendor audit (including inventories) - Mapping - Seasonal Calendars of Food Availability - Market-basket analysis - Photo-elicitation	
	Tool or metric	- MFDI can be adapted to be benchmarked to different food groups, including the MDD-W, the Diet Quality Questionnaire, and national FBDGs. - HEI of Food Supply should be modified for each LMIC to be benchmarked to culturally relevant quantitative FBDGs. - HEI of Food Supply can be modified further to use a vendor-interview methodology to procure quantitative data required for evaluation.
	- MFDI - HEI of Food Supply (adapted version)	
Price	Methods	- Research is needed to modify existing audits and inventories to include culturally relevant food items by food group.
	- Vendor audit - Market-basket analysis	
	Tool or metric	- The CoHD focuses on all food groups recommended as part of selected quantitative FBDGs rather than only on staple foods at the national level. - Research is needed to adapt current metrics that use secondary national price data to include primary data collection in markets. However, collecting market data will be time- and labor-resource-intensive. Additional research and development are needed on lower-cost data-collection techniques using cell phones, price applications, and crowd sourcing.
	- CoHD (adapted version)	
Vendor and Product Characteristics	Method	See Row 2 above, Vendor audit.
	- Vendor audit	
	Tool or metric	- Research in diverse rural areas is needed to better identify market food environments that exist in LMICs. The tool should be adapted to include those market food environments. - The tool also should be adapted to include a range of food items within food groups relevant to understand barriers and opportunities for healthy diets in LMICs.
	- EPOCH (adapted version)	
Marketing and Regulation	Method	See Row 2 above, Vendor audit.
	- Vendor audit	
	Tool or metric	See Row 7 above, EPOCH (adapted version).
	- EPOCH (adapted version)	
Accessibility	Method	- These can be designed to be culturally relevant. - These should be modified to ensure they capture subjective aspects of interactions with the food environment in specific contexts.
	- Consumer surveys/interviews	
	Tool or metric	- This is a low-cost approach in little need of adaptation for measuring physical distance, travel time, transportation, and walking conditions from households to markets.
	- Remoteness of Population - EPOCH (adapted version)	
Affordability	Methods	See Row 2 above, Vendor audit and Market-basket analysis.
	- Vendor audit - Market-basket analysis	
	Tool or metric	See Row 5 above, CoHD.
	- CoHD (adapted version)	
Convenience	Method	See Row 10 above, Consumer surveys/interviews.
	- Consumer surveys/interviews	
	Tool or metric	See Row 7 above, EPOCH (adapted version).
	- EPOCH (adapted version)	
Desirability	Methods	- Sensory surveys require training on attributes being evaluated.
	- Consumer surveys/interviews - Sensory surveys	
	Tool or metric	- Research is needed to develop a market basket of produce that is culturally relevant in different countries through community engagement, including interviews and free listing tasks. - ProDes is limited in measuring food desirability because it only evaluates fruit and vegetables. Research is needed to develop a tool for evaluating food desirability that includes multiple food groups that contribute to a healthy diet.
	- ProDes (adapted version)	

## Supplementary Materials

The following are available online at https://www.mdpi.com/article/10.3390/foods10112728/s1, Table S1: Food Environment Methods, Tools, and Metrics by Food-Environment Dimension, Table S2: Expert Survey: Experiences and Perspectives Regarding Food-Environment Data Collection in LMICs.
