# Supplementary material for: Suitability of Data-Collection Methods, Tools, and Metrics for Evaluating Market Food Environments in Low- and Middle-Income Countries
Source: Foods. 2021 Nov 8;10(11):2728. doi: 10.3390/foods10112728 (PMC8621112; doi:10.3390/foods10112728)
Supplement: Supplementary file 1 [file foods-10-02728-s001.zip › S2 Global Expert Survey - MDPI.pdf]

Perceptions Regarding the Suitability of Food Environment Methods, Tools, and Metrics for Implementation in Rural Contexts in LMICs (version 1)

1. Introduction + Consent Form

This page describes the background information with regards to the survey as well as a consent form for participants to complete.

Greetings! You are being invited to complete this survey as an expert in the areas of food environments, diets, and nutrition. The **goal of this survey** is to elicit the perspectives of experts regarding the suitability of food environment methods, tools, and metrics for implementation in diverse market contexts in low- and middle- income countries (LMICs).

I am carrying out this survey in the capacity as an independent consultant on behalf of **USAID Advancing Nutrition**. USAID Advancing Nutrition is USAID's flagship multi-sectoral nutrition project that draws together global nutrition experience to design, implement, and evaluate programs that address the root causes of malnutrition. However, this survey does not represent the views of any institution or agency.

Your participation in this survey is **voluntary and anonymous.** Your responses will not be identified with you in any way. The survey is grouped into four parts consisting of a total of **20 questions.** The completion of the survey is expected to take approximately **30 to 45 minutes** of your time to complete. ***You may stop your participation in this survey at anytime with no risk to you***.

If you choose to participate in this survey, you will be requested to share your experiences regarding use of various food environment methods, tools, and metrics as well as evaluate specific assessments on the basis of a Likert-scale for specific attributes. This survey is divided into the following four parts:
Part 1: Consent
Part 2: Practices and perceptions regarding food environment **dimensions**
Part 3: Practices and perceptions regarding food environment **methods**
Part 4: Practices and perceptions regarding food environment **tools and metrics**

Please note that there are two versions of this survey. The three versions differ based on Part 4. Version 1 focuses on tools and metrics for evaluating the dimensions of availability. Version 2 focuses on tools and metrics for evaluating the dimensions of marketing, vendor and product characteristics, convenience, and desirability. Version 3 focuses on tools and metrics for evaluating the dimensions of prices and/or affordability. You are requested to select N/A for any tools and metrics that you are not familiar with.

The **results of this survey** will be used to inform a concept note that I am developing for USAID Advancing Nutrition to provide recommendations to USAID Missions for informing the design, implementation, and evaluation of market-based programs to address the root causes of malnutrition. In addition to the concept note, de-identified survey results will potentially be used to develop presentations, protocols, publications, and other material.

The survey will start with a brief consent question regarding your voluntary and anonymous participation. The survey has been approved for exempt status regarding the participation of human subjects by the Institutional Review Board (IRB) of John Snow, Inc. (JSI; IRB #20-25E). If you have any questions, concerns, or suggestions, please do not hesitate to contact me at **selena.ahmed@montana.edu** or the JSI IRB Chair and Research Protections Specialist at IRB@jsi.com.

Thank you for consideration of participating in this survey and informing selection of food environment assessments that are suitable for LMICs.

With best wishes,

Selena Ahmed, PhD
Associate Professor of Sustainable Food Systems
Independent Consultant

1. Do you agree to participate in this survey?

- ◯ **Yes**, I am willing to voluntarily participate in the study outlined above by responding to the following survey questions. I give permission for you to include my anonymous survey responses in your analysis, concept note, and other material (my name and affiliation will not be included).
- ◯ **No**, I would not like to participate in the survey outlined above. I understand my decision will be respected by the research team and I will not face any risk or discrimination based on my decision.

Perceptions Regarding the Suitability of Food Environment Methods, Tools, and Metrics for Implementation in Rural Contexts in LMICs (version 1)

2. Practices and Perceptions Regarding Food Environment Dimensions

Food environments have been classified on the basis of various dimensions. Some researchers / practitioners measure multiple dimensions whereas others focus one specific dimension.

This part of the survey will ask you to respond to five questions regarding your own practice with evaluating food environment dimensions as well as your perceptions of their measurement in rural contexts in low- and middle- income countries (LMICs).

2. What best describes your **profession**?

- ○ Research scientist
- ○ Policy maker
- ○ Field practitioner
- ○ Program evaluator
- ○ Professor
- ○ Industry specialist
- ○ Other (please specify)

[ ]

3. In your own practice, which food environment dimensions do you **generally measure**?

Please select all that apply

- ☐ **Availability**: refers to the presence of a specific food or food groups in a given place
- ☐ **Affordability**: refers to the interaction between prices and individual purchasing power
- ☐ **Price**: refers to the monetary value of food products
- ☐ **Convenience**: refers to the time cost of obtaining, preparing, and consuming a specific food item or group of food items or it may be associated with the proximity of an individual to specific food outlets (stores, markets, restaurants)
- ☐ **Vendor + Product Characteristics:** Vendor characteristics include aspects such as store hours, store type, and other features that may serve to influence consumer food choices such as cleanliness, signage, and overall reputation. Product characteristics include attributes that consumers value, such as food safety, quality, packaging, and processing.
- ☐ **Desirability**: refers to the factors that influence preferences of foods such as sensory attributes including visual appeal, aroma, taste, and texture as well as cultural factors
- ☐ **Marketing + Regulation**: refers to the promotion, advertising, branding, and labeling associated with the marketing and sale of food
- ☐ **Sustainability**: refers to the environmental, socio-economic, and health aspects associated with food items
- ☐ **Accessibility**: pertains to physical and temporal access by individuals to specific foods or food vendors in markets and may relate to car ownership, presence of public transportation, presence of food retail locations within walking distance, and time needed to access the nearest food retail location

Please share any comments regarding your measurements of these food environment dimensions.

[ ]

4. In your own practice, which best refers to your assessment of food environment dimensions?

Please select the option that is most applicable to you

- ◯ I generally implement one food environment assessment (methodology / tool / metric) that is applicable for evaluating multiple food environment dimensions.
- ◯ I generally implement one food environment assessment (methodology / tool / metric) that is applicable for evaluating multiple food environment dimensions **plus** one or more food environment assessments that focus on evaluating specific food environment dimensions.
- ◯ I generally implement one food environment assessment (methodology / tool / metric) for each food environment dimension.
- ◯ Other (please specify)

5. In your own practice, which food environment dimensions do you perceive are the most important to measure for understanding a rural context in LMICs for influencing diets and nutrition?

Please **rank** the following dimensions based on order of perceived importance.

Availability: refers to the presence of a specific food or food groups in a given place

Price: refers to the monetary value of food products

Vendor + Product Characteristics: Vendor characteristics include aspects such as store hours, store type, and other features that may serve to influence consumer food choices such as cleanliness, signage, and overall reputation. Product characteristics include attributes that consumers value, such as food safety, quality, packaging, and processing.

Marketing + Regulation: refers to the promotion, advertising, branding, and labeling associated with the marketing and sale of food

Accessibility: pertains to physical and temporal access by individuals to specific foods or food vendors in markets and may relate to car ownership, presence of public transportation, presence of food retail locations within walking distance, and time needed to access the nearest food retail location

Affordability: refers to the interaction between prices and individual purchasing power

Convenience: refers to the time cost of obtaining, preparing, and consuming a specific food item or group of food items or it may be associated with the proximity of an individual to specific food outlets (stores, markets, restaurants)

Desirability: refers to the factors that influence preferences of foods such as sensory attributes including visual appeal, aroma, taste, and texture as well as cultural factors

Sustainability: refers to the environmental, socio-economic, and health aspects associated with food items

6. This question builds on the previous question and requests for you to take the perspective of a program officer of an agency in an LMIC focused on an evidence-based approach to designing and implementing food environment programs for improving food security.

From the perspective of such a program officer, which food environment dimensions do you perceive are the most important to measure for understanding a rural context in LMICs for influencing diets and nutrition?

Please **rank** the following dimensions based on order of perceived importance.

Availability: refers to the presence of a specific food or food groups in a given place

Price: refers to the monetary value of food products

Vendor + Product Characteristics: Vendor characteristics include aspects such as store hours, store type, and other features that may serve to influence consumer food choices such as cleanliness, signage, and overall reputation. Product characteristics include attributes that consumers value, such as food safety, quality, packaging, and processing.

Marketing + Regulation: refers to the promotion, advertising, branding, and labeling associated with the marketing and sale of food

Accessibility: pertains to physical and temporal access by individuals to specific foods or food vendors in markets and may relate to car ownership, presence of public transportation, presence of food retail locations within walking distance, and time needed to access the nearest food retail location

Affordability: refers to the interaction between prices and individual purchasing power

Convenience: refers to the time cost of obtaining, preparing, and consuming a specific food item or group of food items or it may be associated with the proximity of an individual to specific food outlets (stores, markets, restaurants)

Desirability: refers to the factors that influence preferences of foods such as sensory attributes including visual appeal, aroma, taste, and texture as well as cultural factors

Sustainability: refers to the environmental, socio-economic, and health aspects associated with food items

Perceptions Regarding the Suitability of Food Environment Methods, Tools, and Metrics for Implementation in Rural Contexts in LMICs (version 1)

3. Practices and Perceptions Regarding Food Environment Methods

This part of the survey will ask you to respond to eight questions regarding your perceptions regarding the use of methods corresponding to each food environment dimension in rural contexts in low- and middle- income countries.

Methods as used here are general procedures, techniques, or processes for attaining data.

7. What **characteristics** do you look for in deciding if a specific food environment assessment is **suitable** for implementation in your research / practice?

Please select all that apply.

- ☐ Appropriate for diverse types of food environments (wild, informal markets, formal markets etc)
- ☐ Captures multiple dimensions of the food environment
- ☐ Context specific
- ☐ Culturally relevant
- ☐ Cost effective
- ☐ Objective
- ☐ Other (please specify)
- ☐ Rapid
- ☐ Simple to implement
- ☐ Scalable
- ☐ Translational (findings can easily be used to inform programs and policies)
- ☐ Validated

8. What methods do you think are suitable for measuring the food environment dimension of **AVAILABILITY** in a rural context in LMICs?

Please indicate if you perceive if each method is not suitable, somewhat suitable, and very suitable.

Also, please indicate if you have used each method for the dimension of AVAILABILITY and share any related comments.

	Not suitable	Somewhat suitable	Very suitable
Vendor audit / inventories	◯	◯	◯
Have you used this method (Indicate Y for yes and N for no)?			
Consumer surveys / interviews	◯	◯	◯

	Not suitable	Somewhat suitable	Very suitable
Have you used this method (Indicate Y for yes and N for no)?			
Market basket analysis	○	○	○
Have you used this method (Indicate Y for yes and N for no)?			
Directory analysis (of commercially listed food vendors)	○	○	○
Have you used this method (Indicate Y for yes and N for no)?			
Shelf space measurements	○	○	○
Have you used this method (Indicate Y for yes and N for no)?			
Seasonal calendars of food availability	○	○	○
Have you used this method (Indicate Y for yes and N for no)?			
Freelisting	○	○	○
Have you used this method (Indicate Y for yes and N for no)?			
Participatory mapping	○	○	○
Have you used this method (Indicate Y for yes and N for no)?			
Photo elicitation / Photovoice	○	○	○
Have you used this method (Indicate Y for yes and N for no)?			

9. What methods do you think are suitable for measuring the food environment dimension of **PRICES** in a rural context in LMICs?

Please indicate if you perceive if each method is not suitable, somewhat suitable, and very suitable.

Also, please indicate if you have used each method for the dimension of PRICES and share any related comments.

	Not suitable	Somewhat suitable	Very suitable
Vendor audit / inventories	○	○	○
Have you used this method (Indicate Y for yes and N for no)?			
Consumer surveys / interviews	○	○	○
Have you used this method (Indicate Y for yes and N for no)?			
Market basket analysis	○	○	○
Have you used this method (Indicate Y for yes and N for no)?			

10. What methods do you think are suitable for measuring the food environment dimension of **AFFORDABILITY** in a rural context in LMICs?

Please indicate if you perceive if each method is not suitable, somewhat suitable, and very suitable.

Also, please indicate if you have used each method for the dimension of AFFORDABILITY and share any related comments.

	Not suitable	Somewhat suitable	Very suitable
Consumer surveys / interviews	○	○	○
Have you used this method (Indicate Y for yes and N for no)?			
Market basket analysis	○	○	○
Have you used this method (Indicate Y for yes and N for no)?			

11. What methods do you think are suitable for measuring the food environment dimensions of **VENDOR and PRODUCT PROPERTIES** as well as **MARKETING and REGULATION** in a rural context in LMICs?

Please indicate if you perceive if each method is not suitable, somewhat suitable, and very suitable.

Also, please indicate if you have used each method for the dimensions of VENDOR and PRODUCT PROPERTIES and MARKETING and REGULATION and share any related comments.

	Not suitable	Somewhat suitable	Very suitable
Vendor audit / inventories	○	○	○
Have you used this method (Indicate Y for yes and N for no)?			
Consumer surveys / interviews	○	○	○
Have you used this method (Indicate Y for yes and N for no)?			
Market basket analysis	○	○	○
Have you used this method (Indicate Y for yes and N for no)?			

12. What methods do you think are suitable for measuring the food environment dimensions of **CONVENIENCE** and **ACCESSIBILITY** in a rural context in LMICs?

Please indicate if you perceive if each method is not suitable, somewhat suitable, and very suitable.

Also, please indicate if you have used each method for the dimension of CONVENIENCE and ACCESSIBILITY and share any related comments.

	Not suitable	Somewhat suitable	Very suitable
Consumer surveys / interviews	○	○	○
Have you used this method (Indicate Y for yes and N for no)?			
Market basket analysis	○	○	○
Have you used this method (Indicate Y for yes and N for no)?			
Photo elicitation	○	○	○
Have you used this method (Indicate Y for yes and N for no)?			

13. What methods do you think are suitable for measuring the food environment dimension of **DESIRABILITY** in a rural context in LMICs?

Please indicate if you perceive if each method is not suitable, somewhat suitable, and very suitable.

Also, please indicate if you have used each method for the dimension of DESIRABILITY and share any related comments.

	Not suitable	Somewhat suitable	Very suitable
Consumer surveys / interviews	○	○	○
Have you used this method (Indicate Y for yes and N for no)?			
Sensory surveys	○	○	○
Have you used this method (Indicate Y for yes and N for no)?			

14. Are there **other methods that are not listed** in this section of the survey that you use?

- ○ No
- ○ Maybe / I am not sure
- ○ Yes (please specify)

4. Practices and Perceptions Regarding Food Environment Tools and Metics

This part of the survey will ask you to respond to Likert-scale questions regarding your perceptions of the use of specific tools and metrics corresponding to the specific food environment dimension of AVAILABILITY in rural contexts in low- and middle- income countries.

Tools as used here are specific instruments for data collection that follow a specific methodology and can result in findings for tabulating a metric. Metrics as used here are parameters or indicators used for measurement, comparison or tracking performance.

The following four questions will ask for you to rate the suitability of various food environment tools and metrics for the food environment dimension of **AVAILABILITY** for implementation in rural contexts in LMICs.

Please select N/A if you are not familiar enough with the tool and/or metric for evaluating it .

15. On a scale from 1 to 5, how would you rate the **suitability** of each of the following tools and metrics for measuring the food environment dimension of **AVAILABILITY** for implementation in a rural context in a LMIC?

In this scale, **1 is the least suitable and 5 is the most suitable.** Please select N/A if you are not familiar enough with the tool and/or metric for rating it.

	1 Least suitable	2	3	4	5 Most suitable	N/A
**Healthy Eating Index (HEI) of Food Supply**: The HEI is a measure of diet quality that can be used at the national level to determine if the food supply meets food-based dietary guidelines or other dietary recommendations.	○	○	○	○	○	○
**Market Information Systems (MIS)**: MIS collect and communicate the price of various foods and products. They may also be used to determine the availability of a specific product in the market.	○	○	○	○	○	○
**ProColor Diversity Tool**: The ProColor Diversity Tool is an inventory used to measure the diversity of fruits and vegetables in food environments based on their color (color is used as a proxy for health-promoting phytochemicals).	○	○	○	○	○	○

16. On a scale from 1 to 5, how would you rate the **level of training** that is required to implement the following tools and metrics and, analyze the resulting data for measuring the food environment dimension of **AVAILABILITY** for implementation in a rural context in a LMIC?

In this scale, **1 requires a relatively low level of training and 5 requires a relatively high level of training.** Please select N/A if you are not familiar enough with the tool and/or metric for rating it.

	1 Relatively low level of training	2	3	4	5 Relatively high level of training	N/A
**Healthy Eating Index (HEI) of Food Supply**: The HEI is a measure of diet quality that can be used at the national level to determine if the food supply meets food-based dietary guidelines or other dietary recommendations.	○	○	○	○	○	○
**Market Information Systems (MIS)**: MIS collect and communicate the price of various foods and products. They may also be used to determine the availability of a specific product in the market.	○	○	○	○	○	○
**ProColor Diversity Tool**: The ProColor Diversity Tool is an inventory used to measure the diversity of fruits and vegetables in food environments based on their color (color is used as a proxy for health-promoting phytochemicals).	○	○	○	○	○	○

17. On a scale from 1 to 5, how would you rate the **resource intensity** (resources including time, costs, equipment, material, software for data analysis etc) that is required to implement the following tools and metrics and, analyze the resulting data for measuring the food environment dimension of **AVAILABILITY** for implementation in a rural context in a LMIC?

In this scale, **1 requires a relatively low level of resources and 5 requires a relatively high level of resources.** Please select N/A if you are not familiar enough with the tool and/or metric for rating it.

	1 Relatively low level of resources	2	3	4	5 Relatively high level of resources	N/A
**Healthy Eating Index (HEI) of Food Supply**: The HEI is a measure of diet quality that can be used at the national level to determine if the food supply meets food-based dietary guidelines or other dietary recommendations.	○	○	○	○	○	○
**Market Information Systems (MIS)**: MIS collect and communicate the price of various foods and products. They may also be used to determine the availability of a specific product in the market.	○	○	○	○	○	○
**ProColor Diversity Tool**: The ProColor Diversity Tool is an inventory used to measure the diversity of fruits and vegetables in food environments based on their color (color is used as a proxy for health-promoting phytochemicals).	○	○	○	○	○	○

18. On a scale from 1 to 5, how would you rate how **translatable / operationalizable** the resulting data is for informing evidence-based programs for the following tools and metrics for measuring the food environment dimension of **AVAILABILITY** for implementation in a rural context in a LMIC?

In this scale, **1 means low translational / operationalizable attributes and 5 means relatively high translational / operationalizable attributes.** Please select N/A if you are not familiar enough with the tool and/or metric for rating it.

	1 Low ability for translation to programs	2	3	4	5 High ability for translation to programs	N/A
**Healthy Eating Index (HEI) of Food Supply**: The HEI is a measure of diet quality that can be used at the national level to determine if the food supply meets food-based dietary guidelines or other dietary recommendations.	○	○	○	○	○	○
**Market Information Systems (MIS)**: MIS collect and communicate the price of various foods and products. They may also be used to determine the availability of a specific product in the market.	○	○	○	○	○	○
**ProColor Diversity Tool**: The ProColor Diversity Tool is an inventory used to measure the diversity of fruits and vegetables in food environments based on their color (color is used as a proxy for health-promoting phytochemicals).	○	○	○	○	○	○

19. Are there **any other tools and metrics** that are not listed in this section of the survey that you use?

○ No

○ I am not sure / maybe

○ Yes (please specify)

20. What would you like to see in the development of food environment assessments (methods, tools, and metrics) in the future?

Please use this part of the survey to **share any comments** you have from your own practice and observations.

Thank you for sharing your time, expertise, and perspectives regarding the implementation of food environment assessments in LMICs.

Please contact me for any questions: **selena.ahmed@montana.edu**
